# Supplementary figures and images for: Dramatically diverse Schizosaccharomyces pombe wtf meiotic drivers all display high gamete-killing efficiency
Source: PLoS Genet. 2020 Feb 7;16(2):e1008350. doi: 10.1371/journal.pgen.1008350 (PMC7032740; doi:10.1371/journal.pgen.1008350)

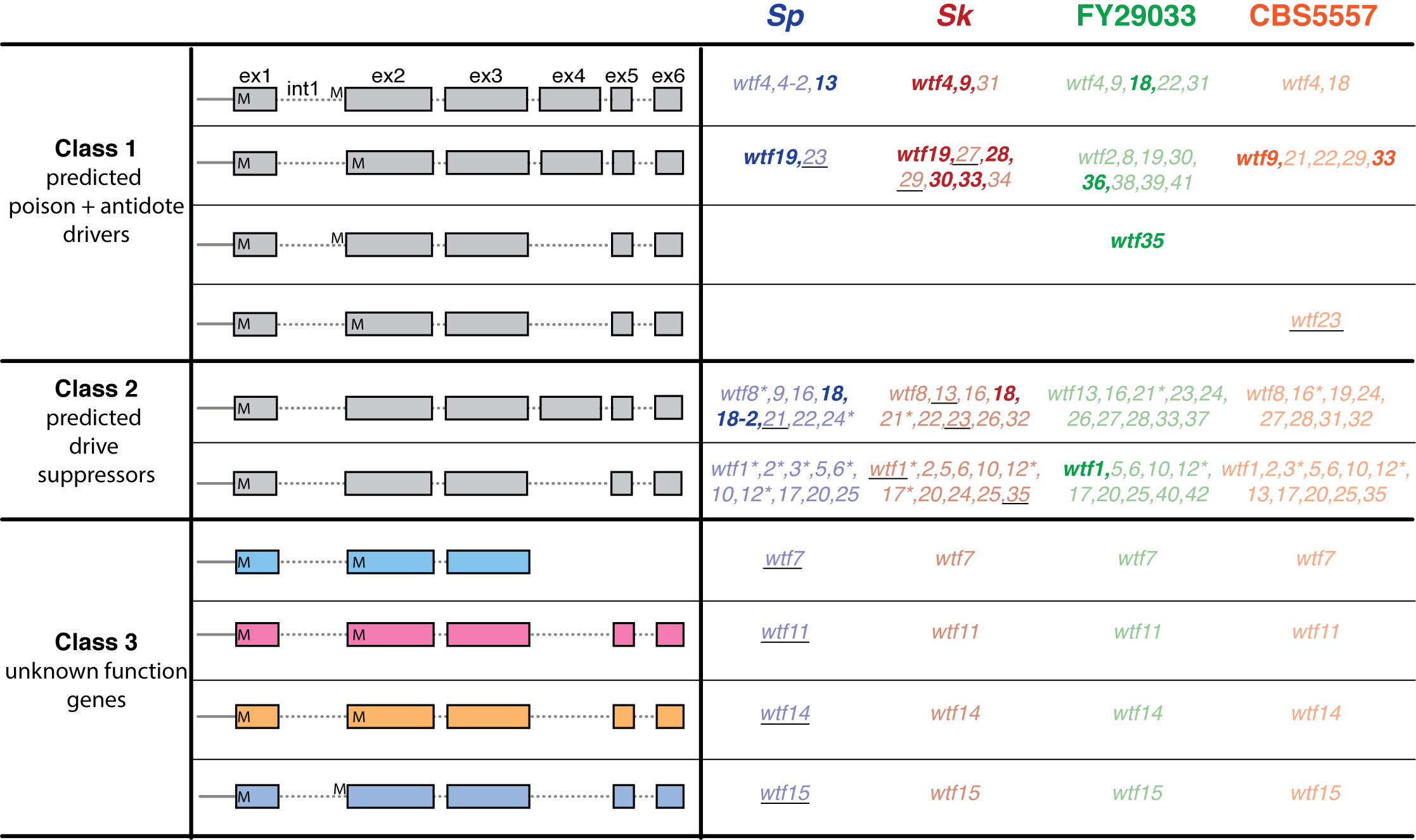

Supplement: S1 Fig — Class 1 is predicted to contain poison-antidote meiotic drivers. Class 2 wtf genes are predicted to be antidote-only genes and suppressors of drive. wtf7, wtf11, wtf14, and wtf15 are all grouped in class 3 solely because they do not look like any other wtf gene in this family, or each other. The wtf genes that have been shown in this study or were previously characterized as meiotic drivers or suppressors are shown in bold [16–18]. The wtf genes that were tested in this study but did not show a meiotic drive or drive suppressor phenotype are underlined. wtf genes with in-frame stop codons are depicted with a “*” next to the gene name and ‘M’ highlights the start codons and the in frame ATG codons near the start of exon 2. (TIF) [file pgen.1008350.s001.tif]

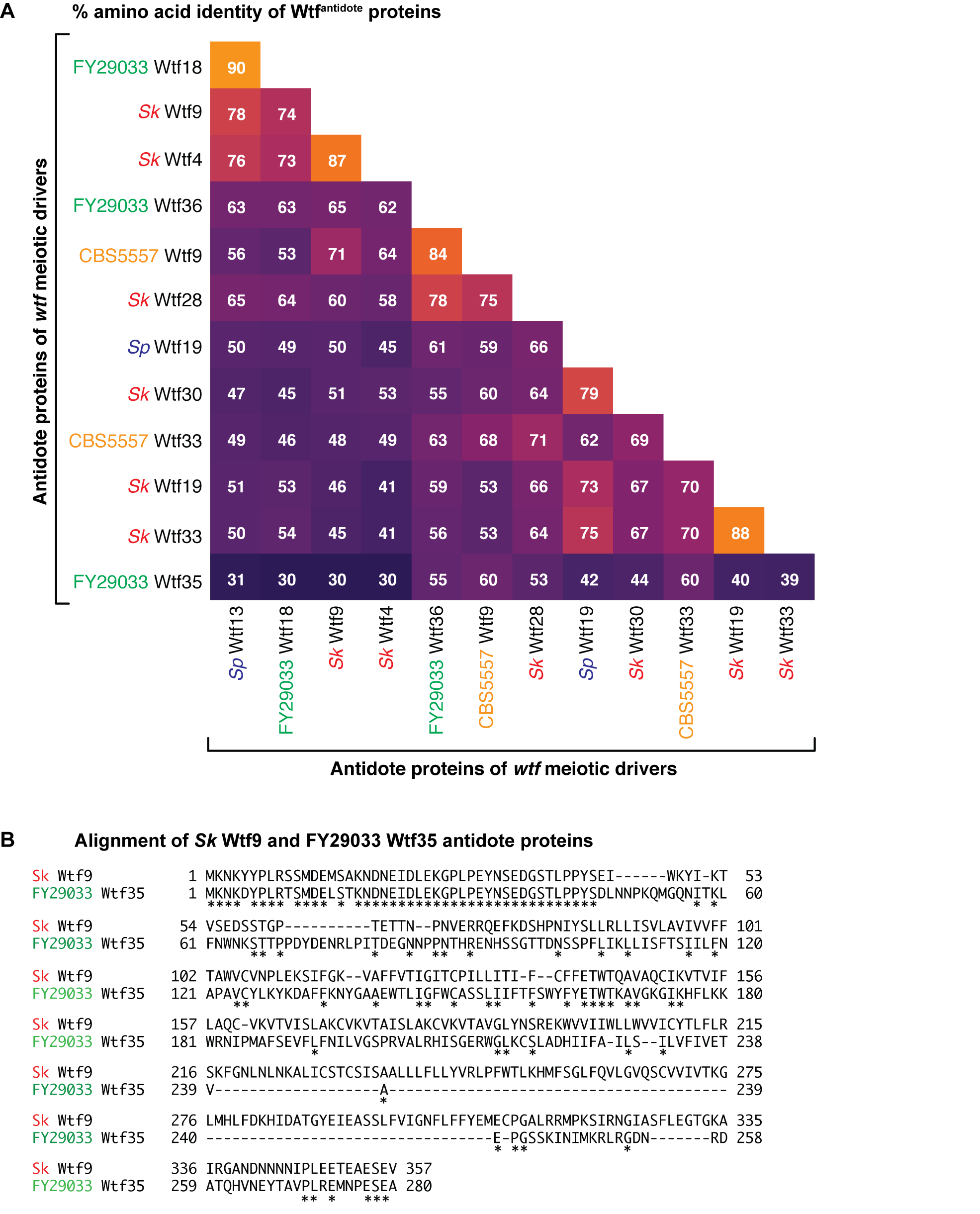

Supplement: S2 Fig — (A) The amino acid identity shared by pairs of Wtfantidote proteins encoded by bona fide meiotic drivers [16–18]. The range of colors corresponds with the ranges of percent amino acid identity between the two compared Wtfantidote proteins. Higher identities are depicted with lighter colors, while lower identities are depicted by darker colors. (B) Manual alignment of the antidotes of Sk Wtf9 and FY29033 Wtf35. Using this method, the percent amino acid identity between these two proteins is 24%. (TIF) [file pgen.1008350.s002.tif]

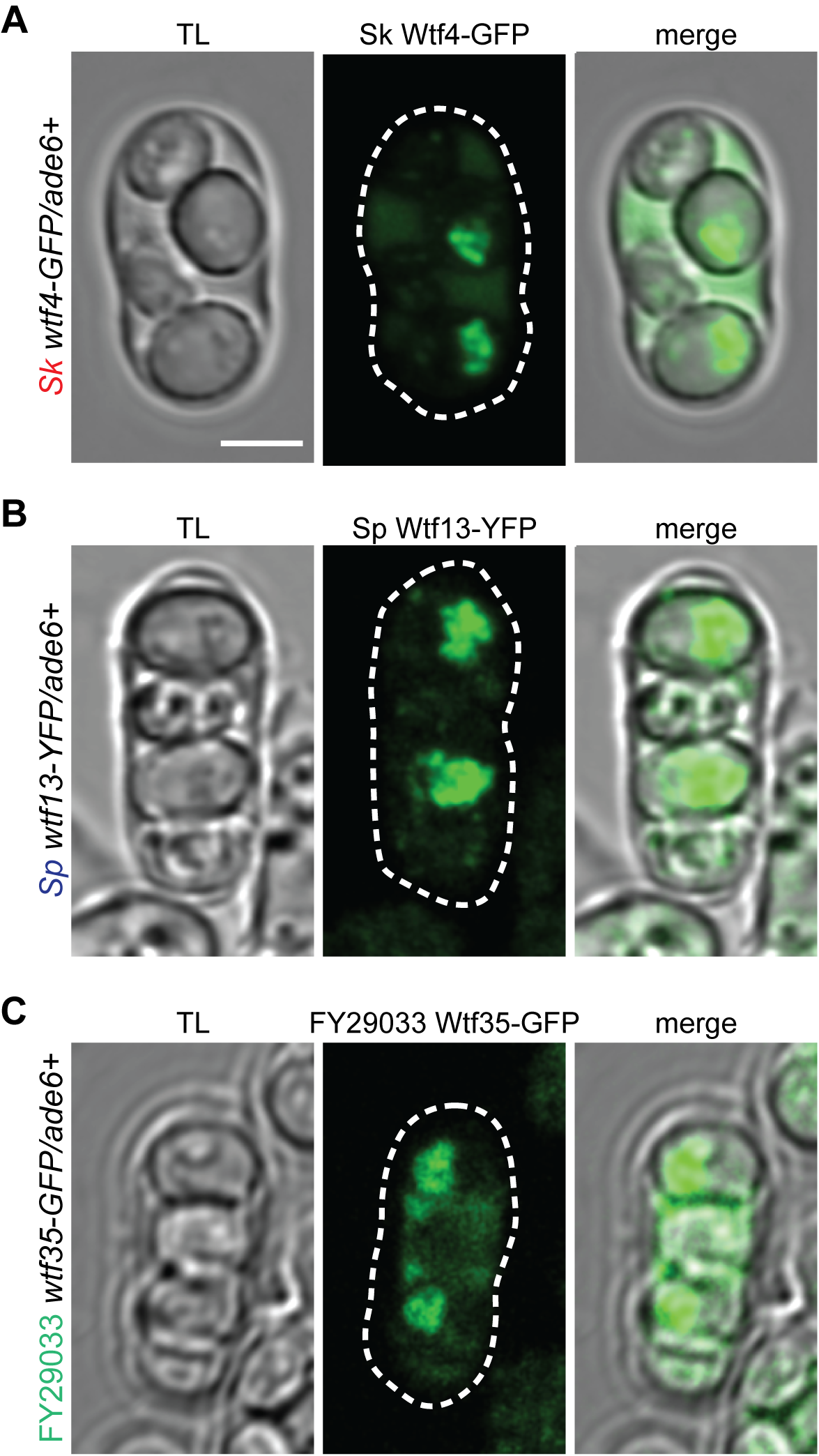

Supplement: S3 Fig — Tetrads showing the localization of (A) Sk Wtf4-GFP, (B) Sp Wtf13-YFP, and (C) FY29033 Wtf35-GFP, and transmitted light (TL) using hemizygous transgenes integrated at ade6 [16, 18]. Brightness and contrast were adjusted differently for each image and the images were smoothed using Gaussian blur. The scale bar represents 3 μm. (TIF) [file pgen.1008350.s003.tif]

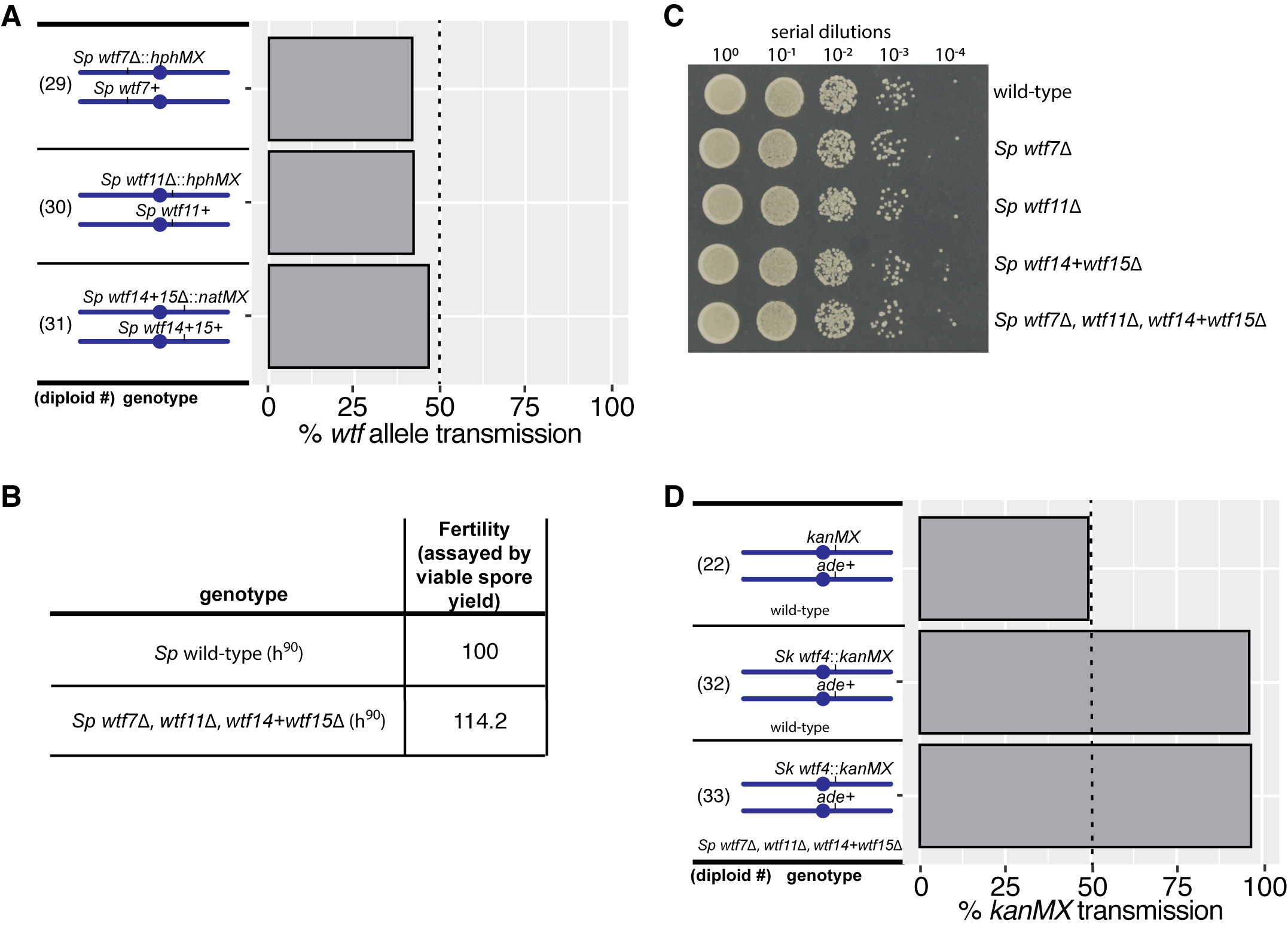

Supplement: S4 Fig — (A) Allele transmission into spores from diploids heterozygous for the class 3 wtf genes. No significant differences in the allele transmission were found using a G-test when compared to the control ura4 locus. (B) Fertility of wild-type and mutant backgrounds (normalized to wild-type). The fertility values were not significantly different (Wilcoxon test). The complete raw data are presented in S2 Table. (C) Serial dilutions of strains with the denoted genotypes were spotted onto YEA plates and grown for 2 days at 32°C. (D) Allele transmission of the Sk wtf4 meiotic driver in a wild-type and mutant background. We found no significant difference between diploid 32 and diploid 33 using a G-test. For (A) and (D), we genotyped more than 200 spore progeny for each diploid and the complete raw data are presented in S1 Table. (TIF) [file pgen.1008350.s004.tif]

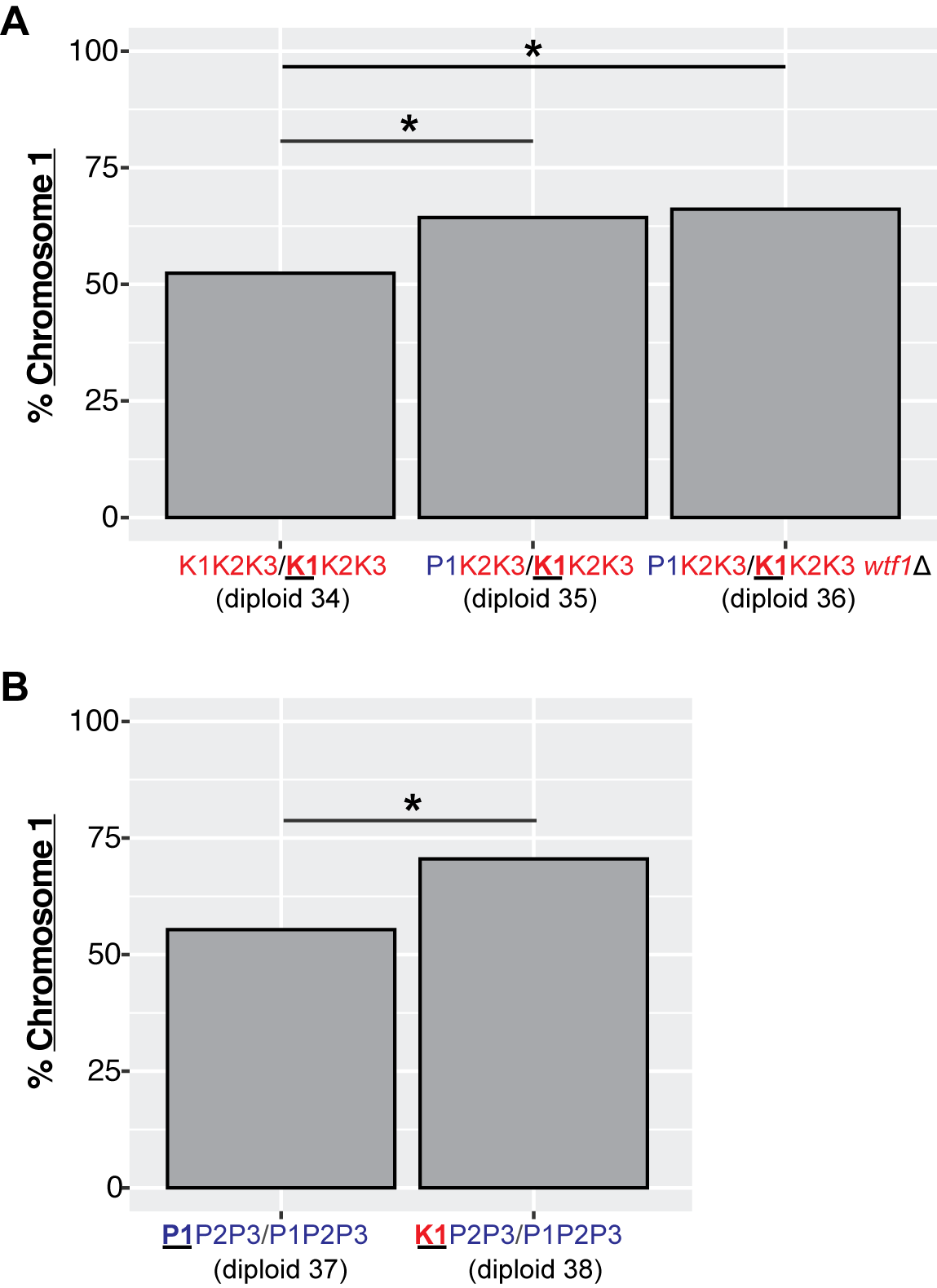

Supplement: S5 Fig — (A) Allele transmission of Sk chromosome 1 (K1) in rec12- diploids. Diploid 35 represents combined data from crosses of three sets of haploid parents. (B) Allele transmission of chromosome 1 from Sp or Sk (P1 or K1, respectively) in rec12- diploids. For A and B, heterozygous markers at the lys1 and/or rec12 loci were used to genotype chromosome 1. At least 400 spore progeny were genotyped for each diploid. * indicates a p-value<0.05 (G-test). The complete raw data are presented in S3 Table. (TIF) [file pgen.1008350.s005.tif]

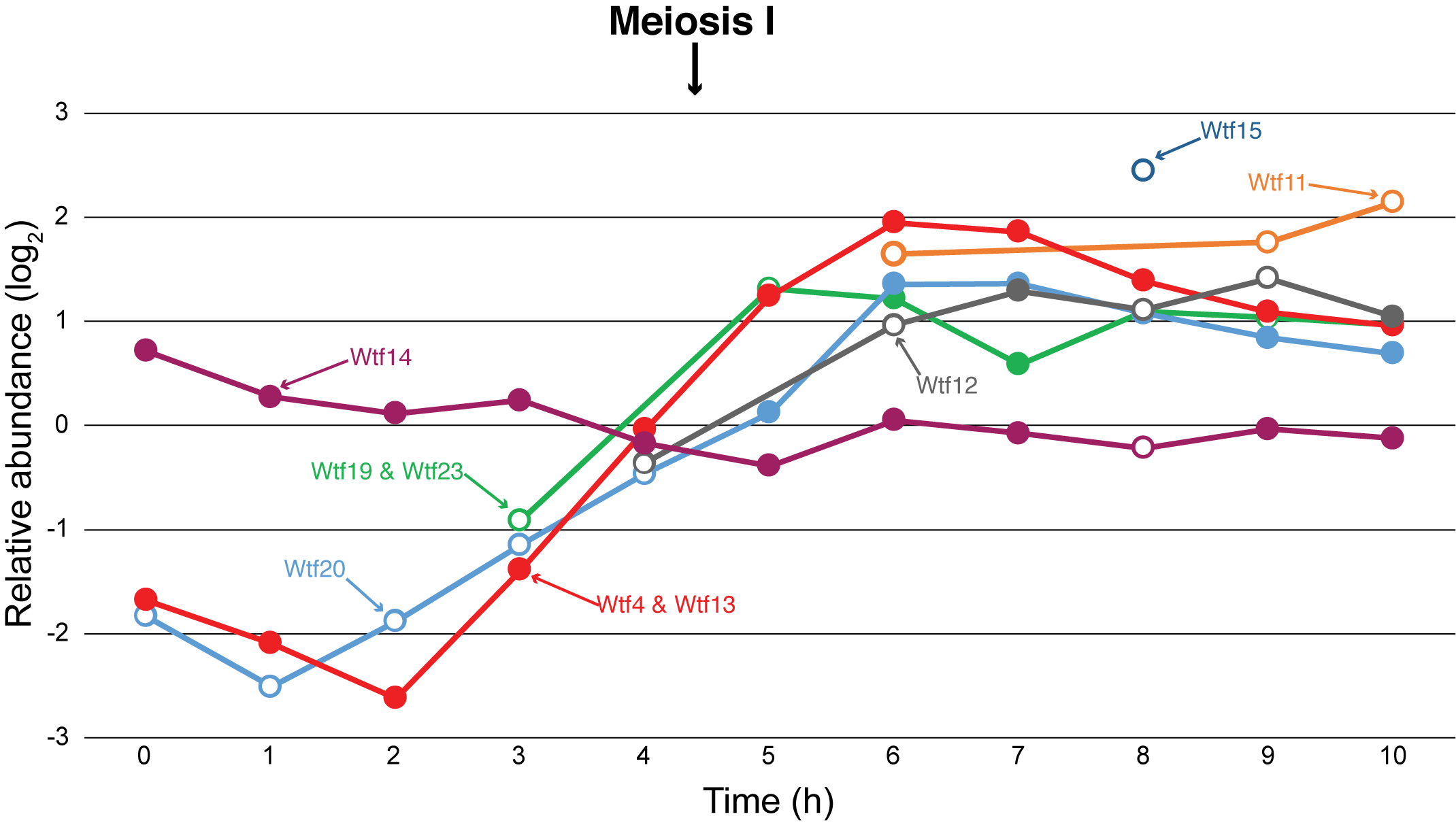

Supplement: S6 Fig — Analysis of Wtf protein levels during meiosis from the data of Krapp et al [29]. In short, Krapp et al prepared a spike-in control by pooling heavy-labeled proteins collected from 0, 3, 5, and 8 hours after meiotic induction. They then compared the constant spike-in control to light-labeled experimental samples collected at the indicated time points [29]. We have plotted the average relative abundance (light-labeled protein to heavy-labeled protein) of the indicated Wtf proteins from three replicate experiments. Peptides for all genes shown were detected in at least two replicate experiments, although not always in each time point. Time points where the protein was detected in less than three experiments are indicated with open circles. Due to the high sequence similarity between Sp Wtf4 and Wtf13 proteins, and Sp Wtf19 and Wtf23 proteins, the data points for these two pairs of proteins were merged. (TIF) [file pgen.1008350.s006.tif]

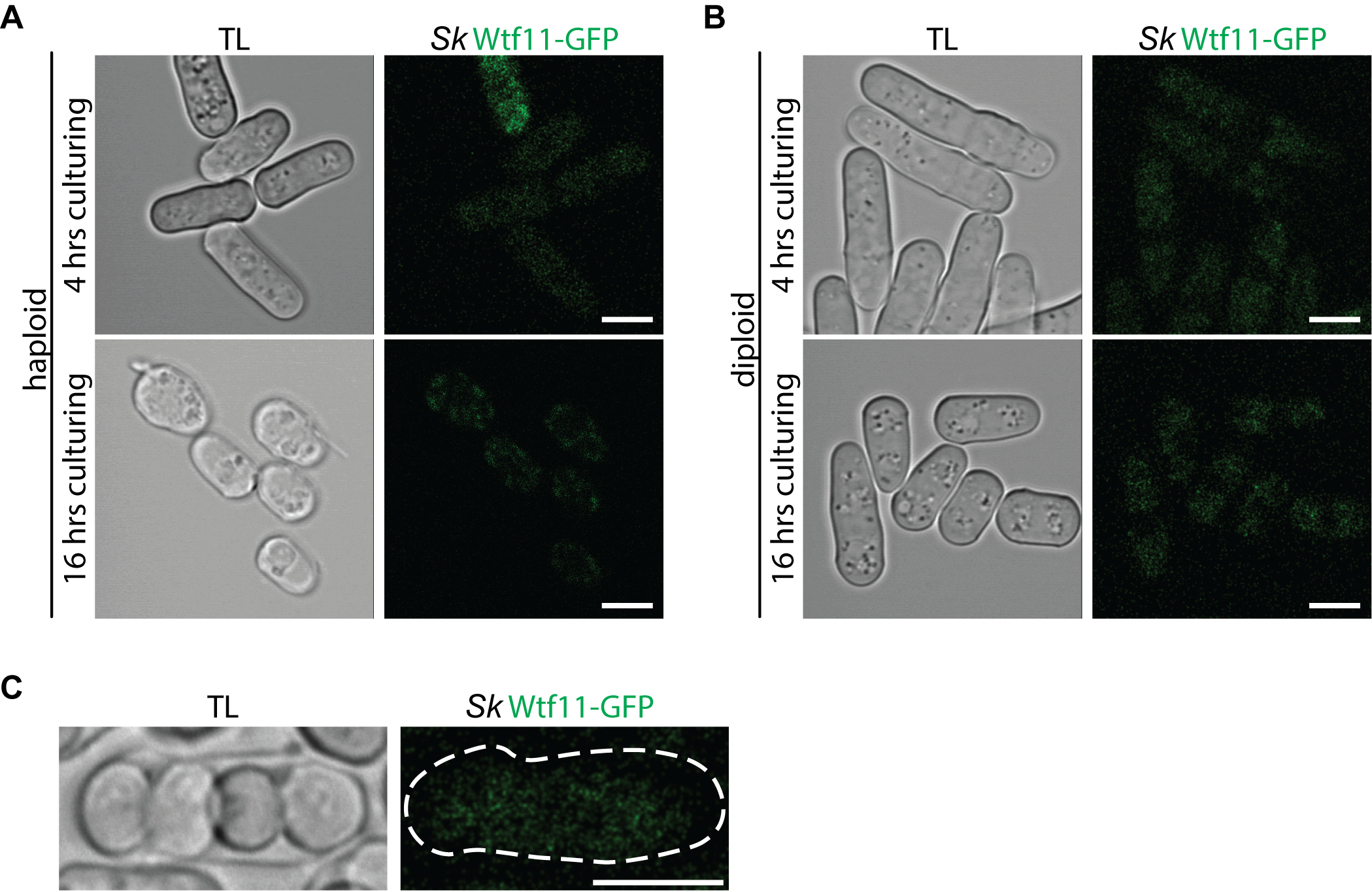

Supplement: S7 Fig — Representative images of cells containing Sk Wtf11-GFP (green) in (A) haploids, (B) heterozygous (wtf11-GFP/ade+) diploids, and (C) a tetrad generated by heterozygous diploids. The GFP signal was not greater than background GFP. We adjusted the brightness and contrast to observe the background. The adjustments were different for each image. We smoothed the images using Gaussian blur. We verified the green autofluorescence via spectral imaging. TL, transmitted light. Scale bar represents 5 μm. (TIF) [file pgen.1008350.s007.tif]

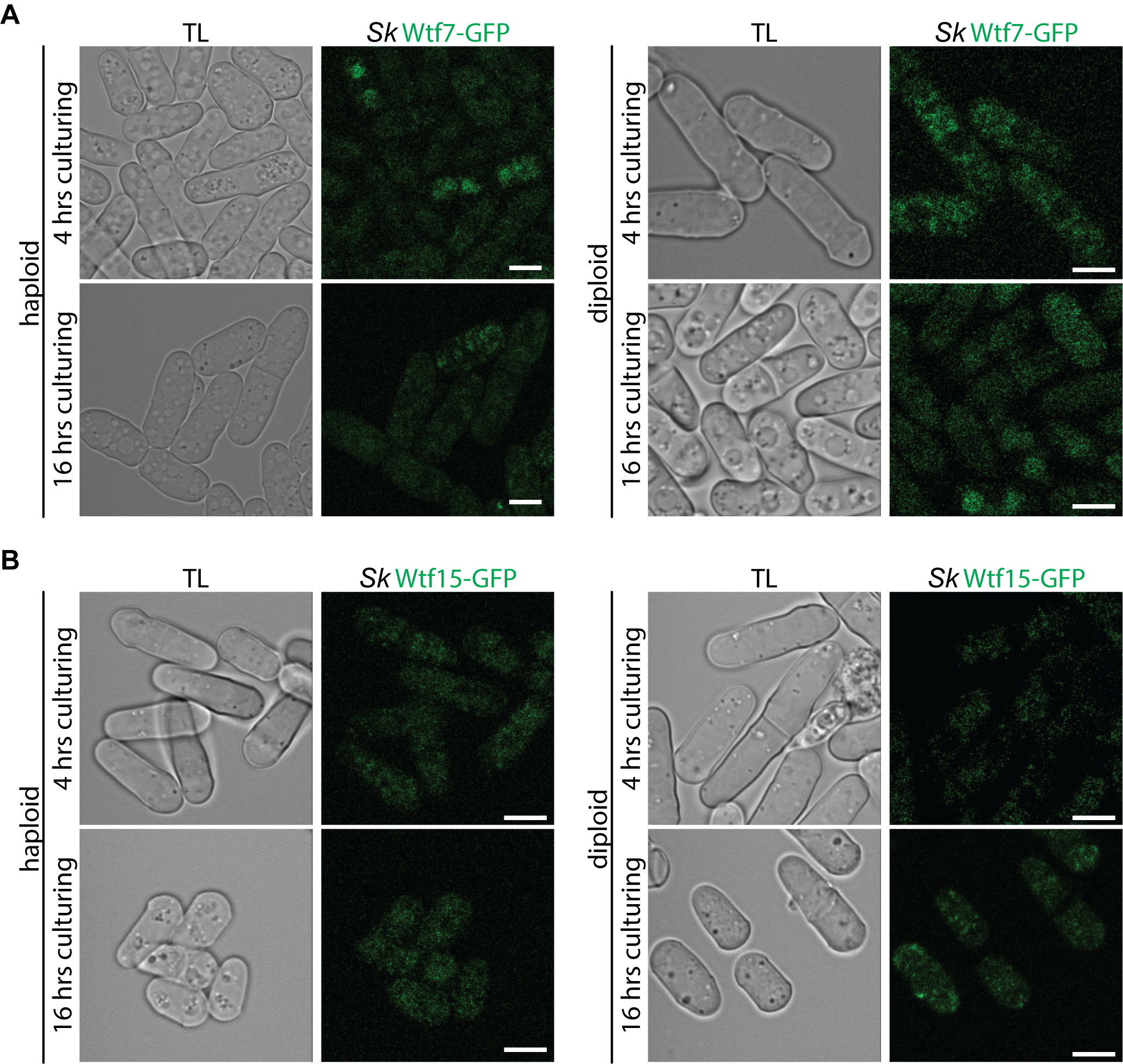

Supplement: S8 Fig — (A) Representative images of Wtf7-GFP (in green) in haploids (left) and heterozygous diploids (right) during logarithmic phase and saturation. (B) Representative images of Wtf15-GFP (in green) in haploids (left) and heterozygous diploids (right) during logarithmic phase and saturation. The GFP signal was not greater than background GFP. We linearly unmixed these images, adjusted the brightness and contrast differently for each image, and smoothed each image using Gaussian blur. The brightness and contrast were adjusted to observe the background. We verified the green autofluorescence via spectral imaging. The scale bar represents 5 μm. TL represents transmitted light. (TIF) [file pgen.1008350.s008.tif]

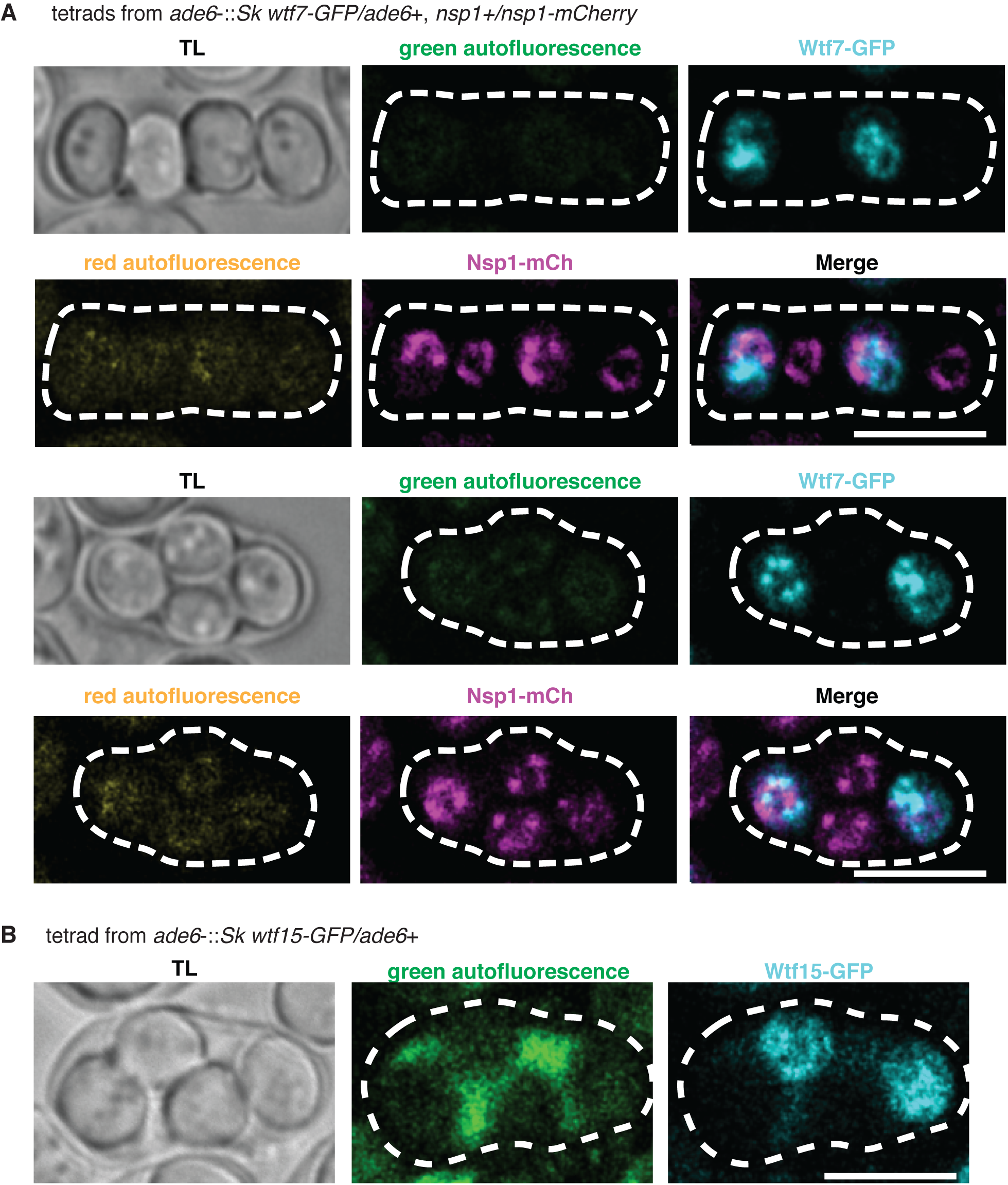

Supplement: S9 Fig — (A) Linear unmixing of the representative images shown in Fig 4A of Sk Wtf7-GFP (cyan) and Nsp1-mCherry (magenta). We normalized the intensities of the green and red autofluorescence to the intensities of the GFP and mCherry signals, respectively. (B) Linear unmixing of the representative image shown in Fig 4B of Sk Wtf15-GFP (cyan) localization. We normalized the intensity of the green autofluorescence to the intensity of the GFP channel. We adjusted the brightness and contrast differently for each image and smoothed them using Gaussian blur. The scale bar represents 5 μm and TL represents transmitted light. (TIF) [file pgen.1008350.s009.tif]

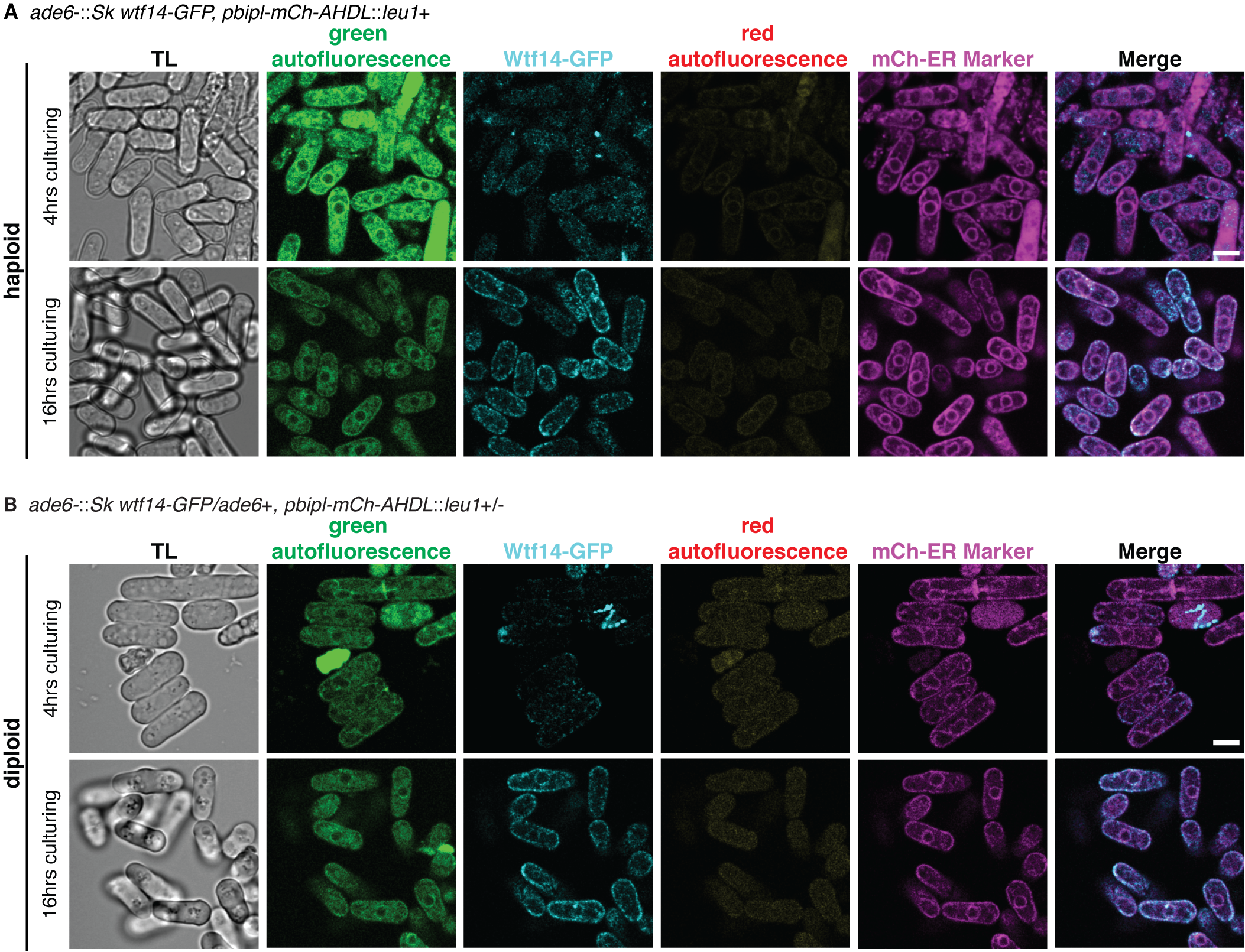

Supplement: S10 Fig — Linear unmixing of (A) haploids and (B) diploids containing Sk Wtf14-GFP (cyan) and mCherry-AHDL (magenta) from the representative images shown in Fig 5A and 5B [30]. We adjusted the intensities of the autofluorescence images to their respective channels. We adjusted brightness and contrast differently for each image and smoothed them using Gaussian blur. TL represents transmitted light. Scale bar represents 5 μm. (TIF) [file pgen.1008350.s010.tif]
