# Supplementary material for: Dramatically diverse Schizosaccharomyces pombe wtf meiotic drivers all display high gamete-killing efficiency
Source: PLoS Genet. 2020 Feb 7;16(2):e1008350. doi: 10.1371/journal.pgen.1008350 (PMC7032740; doi:10.1371/journal.pgen.1008350)
Supplement: S1 Table — Each of the horizontal lines represents the relevant genotype and allele transmission from the indicated diploid into spores. The first column (C1) represents the diploid number, which matches the numbers in Figs 2 and 3, and S4 Fig. In columns C2-C5, the strain number (SZY) and relevant genotype of the haploid parent strains used to determine the allele transmission at the drive locus (ade6 or wtf locus) are shown. Sp, Sk, CBS5557, and FY29033 alleles are labeled in blue, red, yellow, and green, respectively. Columns C6-C8 indicate which phenotypes were followed at the control locus (ura4) and the number of progeny that showed the indicated phenotypes. Columns C9 and C10 indicate the phenotypes that were followed at the drive loci (ade6 or wtf locus) and the number of haploid progeny that exhibited the indicated phenotypes. Some of the progeny inherited both markers from the parent strains at the ade6 locus. DRUGR represents inheritance of either the kanMX4 or hphMX6 marker. G418R signifies inheritance of the kanMX4 marker, HYGR denotes inheritance of the hphMX6 marker, and NATR represents inheritance of the natMX4 marker. Inheritance of these markers confers resistance to G418, Hygromycin B, or Nourseothricin, respectively. The number of the progeny that inherited both markers is presented in column C11 and the percentage of the progeny with this phenotype is shown in column C12. These progeny were excluded from the data presented in Figs 2 and 3, and S3 Fig. Column C13 shows the fraction of the haploid progeny that inherited the genotype of allele 1. Column C14 shows the fraction of the haploid progeny that inherited the genotype of allele 2. Column C15 shows the total progeny assayed excluding the progeny that inherited both genetic markers at ade6. Column C16 shows the total progeny. Column C17 shows the total number of independent diploids assayed for each cross. The last column (C18) shows the p-value calculated by comparing diploids 1–4,7,9,10,12,13,15,17–20 [file pgen.1008350.s011.pdf]

C1	C2	C3	C4	C5	C6	C7	C8	C9	C10	C11	C12	C13	C14	C15	C16	C17	C18
**Diploid #**	**allele 1 SZY#**	**GENOTYPE**	**allele 2 SZY#**	**GENOTYPE**	**ura+**	**ura-**	**% ura+ (control)**	**ade+**	**ade-**	**ade+ DRUG^R**	**% ade+, DRUG^R**	**% allele 1 (excluding ade+, DRUG^R)**	**% allele 2 (excluding ade+, DRUG^R)**	**# progeny assayed (w/o disomes)**	**# progeny assayed**	**# diploids assayed**	**p-value**
	1432	ade6 -:: Sk wtf9 ::kanMX4	44	ade6+	93	72	56.4%	9	156	9	5.2	94.55	5.45	165	174	3	
1	1436	ade6 -:: Sk wtf9 ::hphMX6	44	ade6+	23	29	44.2%	13	39	6	10.3	75.00	25.00	52	58	1	
					116	101	53.5%	22	195	15	6.5	**89.86**	10.14	217	232	4	4.59E-06
	1748	ade6 -:: Sk wtf19 ::hphMX6	643	ade6+	69	41	62.73	1	109	4	3.5	99.09	0.91	110	114	1	
2	1741	ade6 -:: Sk wtf19 ::kanMX4	44	ade6+	122	101	54.71	6	217	37	14.23	97.31	2.69	223	260	2	
					191	142	57.36	7	326	41	10.96	**97.90**	2.10	333	374	3	2.95E-09
3	1257	ade6 -:: Sk wtf30 ::hphMX6	643	ade6+	164	112	59.42	4	272	14	4.8	**98.55**	1.45	276	290	3	1.01E-08
4	1750	ade6 -:: Sk wtf33 ::kanMX4	44	ade6+	108	98	52.43	5	201	9	4.19	**97.57**	2.43	206	215	2	1.95E-07
5	1745	ade6- :: Sp wtf19 ::kanMX4	320	ade6+	131	92	58.74	65	158	9	3.9	**70.85**	29.15	223	232	2	0.0155
6	2431	ade6- :: FY29033 wtf36 ::hphMX6	174	ade6+	130	146	47.1	9	267	10	3.5	**96.74**	3.26	276	286	3	1.38E-06
7	2430	ade6 - :: FY29033 wtf36 ::hphMX6	44	ade6+	105	98	51.7%	9	194	7	3.33	**95.57**	4.43	203	210	3	5.55E-07
	1866	ade6- :: FY29033 wtf18 ::kanMX4	320	ade6+	124	103	54.6%	2	225	5	2.2	99.12	0.88	227	232	4	
8	1867	ade6- :: FY29033 wtf18 ::kanMX4	320	ade6+	126	96	56.8%	5	217	10	4.3	97.75	2.25	222	232	4	
					250	199	55.7%	7	442	15	3.2	**98.4%**	1.6	449	464	8	8.21E-08
9	1860	ade6 -:: FY29033 wtf18 ::kanMX6	643	ade6+	124	99	55.6%	92	131	9	3.9	**58.74**	41.26	223	232	3	0.2098
10	2225	ade6- :: FY29033 wtf35 ::kanMX6	643	ade6+	205	149	57.91	7	347	12	3.3	**98.02**	1.98	354	366	3	1.63E-09
11	2266	ade6- :: FY29033 wtf35 ::hphMX6	320	ade6+	105	105	50	12	198	7	3.23	**94.29**	5.71	210	217	2	1.19E-05
	1764	ade6 -:: Sk wtf27 ::hphMX6	643	ade6+	179	137	56.65	151	165	11	3.4	52.22	47.78	316	327	2	
12	1758	ade6 -:: Sk wtf27 ::kanMX6	44	ade6+	107	87	55.15	102	92	5	2.51	47.42	52.58	194	199	2	
					286	224	56.08	253	257	16	3.04	**50.39**	49.61	510	526	4	0.8431
13	1253	ade6 -:: Sk wtf29 ::hphMX6	643	ade6+	161	66	70.93	107	120	5	2.2	**52.86**	47.14	227	232	4	0.6197
14	1857	ade6- :: Sp wtf23 ::kanMX4	320	ade6+	140	118	54.26	131	127	10	3.7	**49.22**	50.78	258	268	5	0.9445
15	2286	ade6 - :: CBS5557 wtf23 ::kanMX4	643	ade6+	142	117	54.83	108	151	11	4.1	**58.30**	41.70	259	270	4	0.2098
16	2269	ade6- :: CBS5557 wtf23 ::hphMX6	174	ade6+	108	92	54	109	91	9	4.31	**45.50**	54.50	200	209	4	0.693
	1213	ade6 -:: Sk wtf13 ::hphMX6	643	ade6+	103	67	60.59	94	76	4	2.3	44.71	55.29	170	174	2	
17	1211	ade6 -:: Sk wtf13 ::hphMX6	44	ade6+	70	41	63.06	65	46	4	3.5	41.44	58.56	111	115	1	
					173	108	61.57	159	122	8	2.8	**43.42**	56.58	281	289	3	0.3637
	1186	ade6 -:: Sk wtf23 ::kanMX4	643	ade6+	63	45	58.33	57	51	8	6.9	47.22	52.78	108	116	1	
18	1188	ade6 -:: Sk wtf23 ::kanMX4	44	ade6+	114	112	50.44	124	102	6	2.6	45.13	54.87	226	232	2	
					177	157	52.99	181	153	14	4	**45.81**	54.19	334	348	3	0.5795
19	1321	ade6 -:: Sk wtf35 ::hphMX6	643	ade6+	127	98	56.44	94	131	7	3.02	**58.22**	41.78	225	232	3	0.2333
20	2477	ade6- :: FY29033 wtf1 ::hphMX6	44	ade6+	110	95	53.66	101	104	5	2.38	**50.73**	49.27	205	210	2	0.8419
22	925	ade6- ::kanMX4	44	ade6+	233	167	58.25	203	197	27	6.3	**49.250**	50.75	400	427	4	control
23	1516	ade6- ::kanMX4	320	ade6+	103	125	45.18	117	111	4	1.7	**48.68**	51.32	228	232	3	control
	1404	ade6- :: Sp wtf13 ::hphMX6	180	ade6+	173	154	52.9%	13	314	11	3.3	96.02%	3.98	327	338	7	
26	1403	ade6- :: Sp wtf13 ::hphMX6	174	ade6+	89	134	39.9%	6	217	9	3.9	97.31%	2.69	223	232	4	
					262	288	47.6%	19	531	20	3.5	**96.55%**	3.45	550	570	11	1.31E-10
32	887	ade6- :: Sk wtf4 ::kanMX4	44	ade6+	116	124	48.33	9	231	3	1.2	**96.25**	3.75	240	243	2	control
33	3645	ade6- :: Sk wtf4 ::kanMX4	3623	ade6+	NA	NA	NA	12	360	107	22.3	**96.77**	3.23	372	479	4	0.9925
39	3964	ade6 -:: FY29033 wtf35 -GFP::kanMX4	643	ade6+	122	95	56.22	63	154	28	11.4	**70.97**	29.03	217	245	2	0.006128
	**allele 1 SZY#**	**GENOTYPE**	**allele 2 SZY#**	**GENOTYPE**	**ura+**	**ura-**	**% ura+ (control)**	**GEN^R**	**GEN^S**	**GEN^R, HYG^R**	**% GEN^R, HYG^R**	**% allele 1**	**% allele 2**	**# progeny assayed (w/o disomes)**	**# progeny assayed**	**# diploids assayed**	**p-value**
21	890	Sp wtf21 Δ::kanMX4	513	Sp wtf21+	101	103	49.51	114	90	NA	NA	**55.88**	44.12	NA	204	4	0.5111
24	2225	ade6- :: FY29033 wtf35 ::kanMX4	2477	ade6 -:: FY29033 wtf1 ::hphMX6	165	143	53.57	178	130	23	6.95	**57.79**	42.21	308	331	4	0.002168
25	925	ade6 -::kanMX4	1180	ade6 -:: hphMX6	584	513	53.24	452	645	116	9.6	**41.20**	58.80	1097	1213	9	control
	1867	ade6- :: FY29033 wtf18 ::kanMX4	1404	ade6- :: Sp wtf13 ::hphMX6	114	107	51.6%	155	66	8	3.5	70.136	29.86	221	229	4	
27	1866	ade6- :: FY29033 wtf18 ::kanMX4	1404	ade6- :: Sp wtf13 ::hphMX6	119	96	55.3%	133	82	17	7.3	61.860	38.14	215	232	3	
					233	203	53.4%	288	148	25	5.4	**66.06%**	33.94%	436	461	7	0.4228
	**allele 1 SZY#**	**GENOTYPE**	**allele 2 SZY#**	**GENOTYPE**	**ura+**	**ura-**	**% ura+ (control)**	**NAT^R**	**NAT^S**	**HYG^R NAT^R**	**% HYG^R NAT^R**	**% allele 1 (excluding HYG^R NAT^R)**	**% allele 2 (excluding HYG^R NAT^R)**	**# progeny assayed (w/o disomes)**	**# progeny assayed**	**# diploids assayed**	**p-value**
28	3509	ade6 -::natMX4	3954	ade6 Δ::hphMX6	185	146	55.9%	199	132	13	3.8	**60.12%**	39.88%	331	344	6	control
	**allele 1 SZY#**	**GENOTYPE**	**allele 2 SZY#**	**GENOTYPE**	**ura+**	**ura-**	**% ura+ (control)**	**HYG^R**	**HYG^S**			**% allele 1**	**% allele 2**		**# progeny assayed**	**# diploids assayed**	**p-value**
29	2309	Sp wtf7 Δ::hphMX6	643	Sp wtf7+	106	102	50.96	121	87			58.17	**41.83**		208	2	0.2578
	3426	Sp wtf11 Δ::hphMX6	643	Sp wtf11+	65	51	56.034	69	47			59.48	40.52		116	1	
30	3451	Sp wtf11 Δ::hphMX6	2254	Sp wtf11+	64	52	55.172	65	51			56.03	43.97		116	1	
					129	103	55.603	134	98			57.76	**42.24**		232	2	0.09106
	**allele 1 SZY#**	**GENOTYPE**	**allele 2 SZY#**	**GENOTYPE**	**ura+**	**ura-**	**% ura+ (control)**	**NAT^R**	**NAT^S**			**% allele 1**	**% allele 2**		**# progeny assayed**	**# diploids assayed**	**p-value**
31	2856	Sp wtf14+wtf15 Δ::natMX4	345	Sp wtf14, Sp wtf15+	127	96	56.951	119	104			53.36	**46.64**		223	2	0.2188
