# Supplementary material for: Dramatically diverse Schizosaccharomyces pombe wtf meiotic drivers all display high gamete-killing efficiency
Source: PLoS Genet. 2020 Feb 7;16(2):e1008350. doi: 10.1371/journal.pgen.1008350 (PMC7032740; doi:10.1371/journal.pgen.1008350)
Supplement: S2 Table — Each column represents the diploid assayed, which matches the diploid number in Fig 3 and S4 Fig. The first row underneath the diploid number shows the SZY numbers of the haploid parent strains. We present all the viable spore yield values from independent assays. For relative fertility, we normalized diploids 10 and 20 to control diploid 22; diploid 24 to control diploid 25; diploids 8 and 26 to control diploid 23; and diploid 27 to control diploid 28. We previously published the viable spore yield data for diploid 26 [18]. We normalized the average viable spore yield value from the h90 mating of strain SZY3529 (Sp wtf7Δ, wtf11Δ, wtf14+wtf15Δ) to the average viable spore yield value of the wild-type control strain SZY2254. We calculated the p-value comparison using the Wilcoxon test. (PDF) [file pgen.1008350.s012.pdf]

	Figure 3 (VSY values)												
	**Diploid 10**	**Diploid 20**	**Diploid 22**	**Diploid 24**	**Diploid 25**	**Diploid 8**		**Diploid 26**		**Diploid 23**	**Diploid 27**		**Diploid 28**
haploid parents	2225x643	2477x44	925x44	2225x2477	925x1180	1866x320	1867x320	1404x180	1403x174	1516x320	1866x1404	1867x1404	3954x3509
	1.254	1.530	1.996	3.197	2.562	0.933	1.260	1.229	2.378	2.930	1.537	2.368	3.689
	1.546	1.281	1.896	2.142	2.047	1.014	1.531	1.050	1.796	2.684	3.126	2.344	2.665
	1.508	1.800	2.460	3.109	1.960	1.723	1.221	3.000	1.625	1.737	1.490	3.020	2.965
	1.009	2.177	1.980	1.857	2.015	0.845	1.166	2.667	1.850	3.738		1.896	2.945
					2.520					3.400			3.017
					2.557					2.894			3.858
					2.885					2.870			
					4.569								
					4.272								
average	1.329	1.697	2.083	2.576	2.821	1.212		1.949		2.893	2.255		3.190
stdev	0.250	0.384	0.255	0.677	0.961	0.297		0.683		0.626	0.658		0.471
p-value	0.02857	0.2	control	0.9399	control	0.0003108		0.0289		control	0.07343		control
relative fertility	**63.81%**	**81.48%**	**100.00%**	**91.34%**	**100.00%**	**41.88%**		**67.37%**		**100.00%**	**70.68%**		**100.00%**

	Supp Fig 4B (VSY values)	
haploid parent	2254	3529
	1.595	1.463
	1.142	0.882
	1.066	1.005
	0.605	1.414
	0.715	0.922
	0.849	
average	0.995	1.137
stdev	0.357	0.279
p-value	control	0.5368
relative fertility	**100.00%**	**114.24%**
