# Supplementary material for: Dramatically diverse Schizosaccharomyces pombe wtf meiotic drivers all display high gamete-killing efficiency
Source: PLoS Genet. 2020 Feb 7;16(2):e1008350. doi: 10.1371/journal.pgen.1008350 (PMC7032740; doi:10.1371/journal.pgen.1008350)
Supplement: S3 Table — Each of the horizontal lines represents the relevant genotype and allele transmission of the indicated diploid. The first column represents the diploid number, which matches the numbers in S5 Fig. In columns 2–5, the strain number (SZY) and relevant genotype of the haploid parent strains used to determine the allele transmission of chromosome 1 of Sp and Sk. Columns 6 and 7 indicate the number of progeny that exhibited the indicated phenotypes and were followed in chromosome 1 (lys1 or rec12). Column 8 shows the total progeny assayed. Column 9 indicates the percent allele transmission of the Sk chromosome 1. Control diploid 34 (Sk/Sk), diploid 36 (Sp/Sk hybrid wtf1Δ), diploid 37 (Sp/Sp), and diploid 38 (Sp/Sk hybrid) are represented by one pair of haploid parents. Diploid 35 (Sp/Sk hybrid) shows the data from three different pairs of haploid parents. In two of the crosses, we followed chromosome 1 using the lys1 markers. In the third cross, we followed chromosome 1 using the rec12 markers. Column 10 shows the p-value (G-test) calculated by comparing diploid 35 and 36 to control diploid 34, and diploid 38 to control diploid 37. Column 11 shows the total number of diploids assayed for each cross. (PDF) [file pgen.1008350.s013.pdf]

					chr1 allele transmission		# progeny assayed	% allele 2	p value for % allele 2	# diploids assayed
**Diploid #**	**allele 1 SZY#**	**GENOTYPE**	**allele 2 SZY#**	**GENOTYPE**	**lys+**	**lys-**				
**34**	293	*lys1+*	298	*lys1 Δ::kanMX4*	211	232	443	**52.37**	control	2
**35**	3517	*lys1+*	196	*lys1 Δ::kanMX4*	181	283	464	60.99		4
	3518	*lys1+*	196	*lys1 Δ::kanMX4*	126	222	348	63.79		4
	**allele 1 SZY#**	**GENOTYPE**	**allele 2 SZY#**	**GENOTYPE**	**ura-**	**ura+**				
	147	*rec12-117*	298	*rec12 Δ::ura4+*	257	511	768	66.54		3
	**Total Diploid 33**				564	1016	1580	**64.30**	0.02243	11
**36**	147	*rec12-117*	3829	*rec12 Δ::ura4+, wtf1 Δ*	354	690	1044	**66.09**	0.01352	11
**Diploid #**	**allele 1 SZY#**	**GENOTYPE**	**allele 2 SZY#**	**GENOTYPE**	**lys+**	**lys-**				
**37**	3834	*lys1-37*	126	*lys1+*	243	196	439	**55.35**	control	6
**38**	2397	*lys1-37*	150	*lys1+*	282	118	400	**70.50**	0.0299	12
