# Supplementary material for: Dramatically diverse Schizosaccharomyces pombe wtf meiotic drivers all display high gamete-killing efficiency
Source: PLoS Genet. 2020 Feb 7;16(2):e1008350. doi: 10.1371/journal.pgen.1008350 (PMC7032740; doi:10.1371/journal.pgen.1008350)
Supplement: S4 Table — The strain SZY643 contains the wtf18-2 allele [18]. (PDF) [file pgen.1008350.s014.pdf]

Strain	Other name	*S. pombe* isolate	Genotype	Reference
CBS5557	SZY1917	CBS5557	wild-type	
FY29033	SZY1150	FY29033	wild-type	
ySLJ457	SZY3429	*Sp*	*h+, ade6-M216, leu1-32, ura4-D18, his3-D1, nsp1-mCherry::natMX4*	From Sue Jaspersen's lab
SZY13		*Sk*	wild-type	
SZY44	GP745	*Sp*	*h-, lys4-95*	from Gerry Smith's lab
SZY126	GP497	*Sp*	*h-, ade6-52, rec12-117*	from Gerry Smith's lab
SZY147		*Sp*/*Sk* hybrid	*Chr1 Sp, Chr2 and 3 Sk; h90, rec12-117, lys4 △::kanMX4, ura4 △::kanMX4*	27
SZY150		*Sp*/*Sk* hybrid	*Chr1 Sk, Chr2 and 3 Sp; h90, rec12 △::ura4+, ade6-M26, ura4-294*	27
SZY174		*Sk*	*h90, his5 △::natMX4*	27
SZY180		*Sk*	*h90, lys1 △::kanMX4*	27
SZY196		*Sk*	*h90, lys1 △::kanMX4, ura4 △::kanMX4 , rec12 △::ura4+*	27
SZY293		*Sk*	*h90, ade6 △::hphMX6, ura4 △::kanMX4 , rec12 △::ura4+*	27
SZY298		*Sk*	*h90, lys1 △::kanMX4, ura4 △::kanMX4 , his5 △::natMX4 , rec12 △::ura4+*	27
SZY320		*Sk*	*h90, ura4 △::natMX4*	27
SZY345	GP1682	*Sp*	*h+, lys4-95*	from Gerry Smith's lab
SZY481	GP283	*Sp*	*h+, his5-303*	from Gerry Smith's lab
SZY513		*Sp*	*h? (+ or -), his5-303, lys1-37, ade6-M210*	this work
SZY643		*Sp*	*h90, leu1-32, ura4-D18*	16
SZY661		*Sk*	*h90, ura4 △::natMX4, leu1 △::hphMX6*	16
SZY887		*Sp*	*h90, leu1-32, ura4-D18, ade6-::Sk wtf4::kanMX4::ade6-*	16
SZY890		*Sp*	*h90, leu1-32, ura4-D18, wtf21 △::kanMX4*	this work
SZY925		*Sp*	*h90, leu1-32, ura4-D18, ade6-::kanMX4::ade6-*	16
SZY960		*Sp*	*h90, leu1-32, ura4-D18, ade6-::Sk wtf4-GFP::kanMX4::ade6-*	16
SZY1030		*Sp*	*h?, hht1-RFP::kanMX6, lys1-37*	16
SZY1180		*Sp*	*h-; lys4-95; ade6-::hphMX6::ade6-*	this work
SZY1186		*Sp*	*h-, lys4-95, ade6-::Sk wtf23::kanMX4::ade6-*	this work
SZY1188		*Sp*	*h90, leu1-32, ura4-D18, ade6-::Sk wtf23::kanMX4::ade6-*	this work
SZY1211		*Sp*	*h90, leu1-32, ura4-D18, ade6-::Sk wtf13::hphMX6::ade6-*	this work
SZY1213		*Sp*	*h-, lys4-95, ade6-::Sk wtf13::hphMX6::ade6-*	this work
SZY1253		*Sp*	*h-, lys4-95, ade6-::Sk wtf29::hphMX6::ade6-*	this work
SZY1257		*Sp*	*h-, lys4-95, ade6-::Sk wtf30::hphMX6::ade6-*	this work
SZY1321		*Sp*	*h-, lys4-95, ade6-::Sk wtf35::hphMX6::ade6-*	this work
SZY1403		*Sk*	*h90, ura4 △::natMX4, ade6-::Sp wtf13::hphMX6::ade6-*	18
SZY1404		*Sk*	*h90, ura4 △::natMX4, ade6-::Sp wtf13::hphMX6::ade6-*	18
SZY1432		*Sp*	*h90, leu1-32, ura4-D18, ade6-::Sk wtf9::kanMX4::ade6-*	this work
SZY1436		*Sp*	*h90, leu1-32, ura4-D18, ade6-::Sk wtf9::hphMX6::ade6-*	this work
SZY1516		*Sk*	*h90, his5 △::natMX4, ade6-:::kanMX4::ade6-*	this work
SZY1554		*Sk*	*h90, his5 Δ::natMX4, ade6-::Sp wtf13-YFP::kanMX4::ade6-*	18
SZY1741		*Sp*	*h90, leu-32, ura4-D18, ade6-::Sk wtf19::kanMX4::ade6-*	this work
SZY1745		*Sk*	*h90, his5 △::natMX4, ade6-::Sp wtf19::kanMX4::ade6-*	this work
SZY1748		*Sp*	*h-, lys4-95, ade6-::Sk wtf19::hphMX6::ade6-*	this work
SZY1750		*Sp*	*h90, leu-32, ura4-D18, ade6-::Sk wtf33::kanMX4::ade6-*	this work
SZY1758		*Sp*	*h90, leu-32, ura4-D18, ade6-::Sk wtf27::kanMX4::ade6-*	this work
SZY1764		*Sp*	*h-, lys4-95, ade6-::Sk wtf27::hphMX6::ade6-*	this work
SZY1857		*Sk*	*h90, his5 △::natMX4, ade6-::Sp wtf23::kanMX4::ade6-*	this work
SZY1860		*Sp*	*h-, lys4-95, ade6-:: FY29033 wtf18::kanMX4::ade6-*	this work
SZY1866		*Sk*	*h90, his5 △::natMX4, ade6-:: FY29033 wtf18::kanMX4::ade6-*	this work
SZY1867		*Sk*	*h90, his5 △::natMX4, ade6-:: FY29033 wtf18::kanMX4::ade6-*	this work
SZY2225		*Sp*	*h-, lys4-95, ade6-:: FY29033 wtf35::kanMX4::ade6-*	this work
SZY2253		*Sp*	*h-, lys4-95, wtf21 △::kanMX4*	this work
SZY2254		*Sp*	*h90, lys4-95*	this work
SZY2266		*Sk*	*h90, his5 △::natMX4, ade6-:: FY29033 wtf35::hphMX6::ade6-*	this work
SZY2269		*Sk*	*h90, ura4 △::natMX4, ade6-:: CBS5557 wtf23::hphMX6::ade6-*	this work
SZY2286		*Sp*	*h-, lys4-95, ade6-:: CBS5557 wtf23::kanMX4::ade6-*	this work
SZY2309		*Sp*	*h-, lys4-95, Sp wtf7 △::hphMX6*	this work
SZY2310		*Sp*	*h90, leu1-32, ura4-D18, Sp wtf7 △::hphMX6*	this work
SZY2336		*Sp*	*h90, leu1-32, ura4-D18, Sp wtf7 △::natMX4*	this work
SZY2397		*Sp*	*h+; lys1-37, rec12-169::3HA6His-kanMX6, ura4 △::natMX4*	this work
SZY2430		*Sp*	*h90, leu1-32, ura4-D18, ade6-:: FY29033 wtf36::hphMX6::ade6-*	this work
SZY2431		*Sk*	*h90, ura4 △::natMX4, ade6-:: FY29033 wtf36::hphMX6::ade6-*	this work
SZY2477		*Sp*	*h90, leu1-32, ura4-D18, ade6-:: FY29033 wtf1::hphMX6::ade6-*	this work
SZY2854		*Sp*	*h90, leu1-32, ura4-D18, Sp wtf7 △::natMX4, Sp wtf11 △::hphMX6*	this work
SZY2855		*Sp*	*h90, leu1-32, ura4-D18, Sp wtf7 △::natMX4, Sp wtf11 △::hphMX6*	this work
SZY2856		*Sp*	*h90, leu1-32, ura4-D18, Sp wtf14 + Sp wtf15 △::natMX4*	this work
SZY2857		*Sp*	*h90, leu1-32, ura4-D18, Sp wtf14 + Sp wtf15 △::natMX4*	this work
SZY2943		*Sp*	*h90, leu1-32, ura4-D18, ade6-::Sk wtf15-GFP::kanMX4::ade6-, his5 △::ade6+*	this work
SZY2944		*Sp*	*h90, leu1-32, ura4-D18, ade6-::Sk wtf15-GFP::kanMX4::ade6-, his5 △::ade6+*	this work
SZY3111		*Sp*	*h90, his5 △::ade6+, leu1-32, ura4-D18, ade6-::Sk wtf14-GFP::kanMX4::ade6-, pbipl-mCherry-AHDL::leu1+*	this work
SZY3426		*Sp*	*h?, lys4-95, Sp wtf11 △::hphMX6*	this work
SZY3427		*Sp*	*h?, lys4-95, Sp wtf14 + Sp wtf15 △::natMX4*	this work
SZY3444		*Sp*	*h90, leu1-32, ura4-D18, Sp wtf14 + Sp wtf15 Δ::kanMX4*	this work
SZY3448		*Sp*	*h90, leu1-32, ura4-D18, Sp wtf14 + Sp wtf15 △::CaURA3MX*	this work
SZY3451		*Sp*	*h-, leu-32, ura4-D18, Sp wtf11 △::hphMX6*	this work
SZY3495		*Sp*	*h-, lys4-95, ade6-::Sk wtf7-GFP::kanMX4::ade6-, his5 △::ade6+*	this work
SZY3509		*Sk*	*h90, lys4△::kanMX4, ade6-::natMX4::ade6-*	this work
SZY3517		*Sp*/*Sk* hybrid	*Chr1 Sp, Chr2 and 3 Sk; h90, rec12-117, ura4 △::kanMX4, his5 △::natMX4*	this work
SZY3518		*Sp*/*Sk* hybrid	*Chr1 Sp, Chr2 and 3 Sk; h90, rec12-117, ura4 △::kanMX4, his5 △::natMX4*	this work
SZY3529		*Sp*	*h90, lys4-95, Sp wtf7 △::natMX4, Sp wtf11 △::hphMX6, Sp wtf14 + Sp wtf15 △::kanMX4*	this work
SZY3532		*Sp*	*h-, lys4-95, Sp wtf7 △::natMX4, Sp wtf11 △::hphMX6, Sp wtf14 + Sp wtf15 △::kanMX4*	this work
SZY3541		*Sp*	*h? (+ or -), leu1-32, ura4-D18, nsp1-mCherry::natMX4*	this work
SZY3563		*Sp*	*h90, leu1-32, ura4-D18, Sp wtf14 + Sp wtf15 △::CaURA3MX, Sp wtf7 △::natMX4, Sp wtf11 △::hphMX6*	this work
SZY3623		*Sp*	*h?, lys4-95, Sp wtf14 + Sp wtf15 △::CaURA3MX, Sp wtf7 △::natMX6, Sp wtf11 △::hphMX6, (ura4-D18?)*	this work
SZY3626		*Sp*	*h90, lys4-95, ade6-::Sk wtf11-GFP::kanMX4::ade6-*	this work
SZY3645		*Sp*	*h90, leu1-32, ura4-D18, Sp wtf14 + Sp wtf15 △::CaURA3MX, Sp wtf7 △::natMX4, Sp wtf11 △::hphMX6, ade6-::Sk wtf4::kanMX4::ade6-*	this work
SZY3725		*Sp*	*h90, lys4-95, ade6-::Sk wtf11-GFP::kanMX4::ade6-, his5 △::ade6+*	this work
SZY3829		*Sk*	*h90, ura4 △::kanMX4, lys1 △::kanMX4, his5 △::natMX4, rec12 △::ura4+, Sk wtf1 △::hphMX6*	this work
SZY3834		*Sp*	*h90, leu1-32, rec12-117, ura4-x, lys1-37*	this work
SZY3954		*Sk*	*h90, ade6 Δ::hphMX6, ura4-::kanMX4::ura4-*	this work
SZY3964		*Sp*	*h90, lys4-95, ade6-::FY29033 wtf35-GFP::kanMX4::ade6-*	this work
