# Supplementary material for: Dramatically diverse Schizosaccharomyces pombe wtf meiotic drivers all display high gamete-killing efficiency
Source: PLoS Genet. 2020 Feb 7;16(2):e1008350. doi: 10.1371/journal.pgen.1008350 (PMC7032740; doi:10.1371/journal.pgen.1008350)
Supplement: S5 Table — Column 1 lists the wtf gene cloned into each vector. Column 2 denotes the isolate origin of each wtf gene in column 1. The DNA templates and oligos used in the PCR reactions to amplify the wtf alleles are shown in columns 3 and 4, respectively. We digested each of the amplified fragments with the enzymes reported in column 5 and then integrated into the target site listed in column 6. The strain number of each of the plasmids that we generated is reported in column 7. The description of each plasmid can be found in S6 Table. (PDF) [file pgen.1008350.s015.pdf]

***wtf* allele**	***S. pombe* isolate**	**DNA template (gDNA)**	**PCR oligos used**	**PCR fragment digested with restriction enzyme**	**target site**	**Resulting plasmid number**
wtf19	*Sp*	SZY643	1129 + 1130	SacI	SacI site of pSZB188	pSZB507
wtf23	*Sp*	SZY44	890 + 891	SacI	SacI site of pSZB188	pSZB372
wtf9	*Sk*	SZY13	1033+1034	EcoRV	EcoRV site of pSZB188	pSZB466
wtf9	*Sk*	SZY13	1033+1034	EcoRV	EcoRV site of pSZB386	pSZB468
wtf13	*Sk*	SZY180	2260+2261	SpeI	SpeI site of pSZB386	pSZB399
wtf14	*Sk*	SZY180	881+882	SacI	SacI site of pSZB188	pSZB378
wtf19	*Sk*	SZY661	1129+1133	SacI	SacI site of pSZB188	pSZB511
wtf19	*Sk*	SZY661	1129+1133	SacI	SacI site of pSZB386	pSZB512
wtf23	*Sk*	SZY180	890 + 891	SacI	SacI site of pSZB188	pSZB379
wtf27	*Sk*	SZY661	1134+1135	SpeI	SpeI site of pSZB188	pSZB516
wtf27	*Sk*	SZY661	1134+1135	SpeI	SpeI site of pSZB386	pSZB519
wtf29	*Sk*	SZY180	975+976	SpeI	SpeI site of pSZB386	pSZB409
wtf30	*Sk*	SZY180	977+978	SpeI	SpeI site of pSZB386	pSZB410
wtf33	*Sk*	SZY661	1131+1132	SacI	SacI site of pSZB188	pSZB514
wtf35	*Sk*	SZY13	1035+1036	SacI	SacI site of pSZB386	pSZB462
wtf18	FY29033	FY29033	1039+1202	SacI	SacI site of pSZB188	pSZB661, pSZB662
wtf35	FY29033	FY29033	1036+1349	SacI	SacI site of pSZB188	pSZB788
wtf35	FY29033	FY29033	1036+1349	SacI	SacI site of pSZB387	pSZB800
wtf36	FY29033	FY29033	1351+1593	SacI	SacI site of pSZB387	pSZB852, pSZB853
wtf23	CBS5557	CBS5557	890+891	SacI	SacI site of pSZB188	pSZB810
wtf23	CBS5557	CBS5557	890+891	SacI	SacI site of pSZB387	pSZB812
