# Supplementary material for: Dramatically diverse Schizosaccharomyces pombe wtf meiotic drivers all display high gamete-killing efficiency
Source: PLoS Genet. 2020 Feb 7;16(2):e1008350. doi: 10.1371/journal.pgen.1008350 (PMC7032740; doi:10.1371/journal.pgen.1008350)
Supplement: S6 Table — (PDF) [file pgen.1008350.s016.pdf]

Plasmids	short description	reference
pFA6	contains *kanMX4*	59
pAG25	contains *natMX4*	60
pAG32	contains *hphMX6*	60
pKT127	contains yEGFP	65
pFA6-mTurq2-URA3MX	contains mTurq2 and CaURA3MX	63
pMZ222	pART1 with Cas9 *(adh1* promoter)	64
pMZ283	pUR19 with *rrk1* -gRNA (CspCI targeting sequence placeholder)	64
pSZB188	derivative of pFA6 that integrates at *ade6* , yielding *ade6* -	16
pSZB189	pSZB188 with *Sk wtf4* cloned into SacI site	16
pSZB197	pMZ283 with gRNA targeting *wtf21* cloned into the CspCI site	this work
pSZB331	derivative of pFA6 that integrates at *ura4* , yielding *ura4-*	this work
pSZB372	pSZB188 with *Sp wtf23* cloned into SacI site	this work
pSZB378	pSZB188 with *Sk wtf14* cloned into SacI site	this work
pSZB379	pSZB188 with *Sk wtf23* cloned into SacI site	this work
pSZB386	derivative of pAG32 that integrates at *ade6* , yielding *ade6* -	18
pSZB387	derivative of pAG32 that integrates at *ade6* , yielding *ade6* -	18
pSZB399	pSZB386 with *Sk wtf13* cloned into SpeI site	this work
pSZB409	pSZB386 with *Sk wtf29* cloned into SpeI site	this work
pSZB410	pSZB386 with *Sk wtf30* cloned into SpeI site	this work
pSZB462	pSZB386 with *Sk wtf35* cloned into SacI site	this work
pSZB466	pSZB188 with *Sk wtf9* cloned into the EcoRV site	this work
pSZB468	pSZB386 with *Sk wtf9* cloned into the EcoRV site	this work
pSZB507	pSZB188 with *Sp wtf19* cloned into SacI site	this work
pSZB511	pSZB188 with *Sk wtf19* cloned into SacI site	this work
pSZB512	pSZB386 with *Sk wtf19* cloned into SacI site	this work
pSZB514	pSZB188 with *Sk wtf33* cloned into SacI site	this work
pSZB516	pSZB188 with *Sk wtf27* cloned into SpeI site	this work
pSZB519	pSZB386 with *Sk wtf27* cloned into SpeI site	this work
pSZB661	pSZB188 with FY29033 *wtf18* cloned into the SacI site	this work
pSZB662	pSZB188 with FY29033 *wtf18* cloned into the SacI site	this work
pSZB691	pSZB188 with *Sk wtf7-GFP* cloned into SacI site	this work
pSZB696	pSZB188 with *Sk wtf14-GFP* cloned into SacI site	this work
pSZB698	pSZB188 with *Sk wtf15-GFP* cloned into SacI site	this work
pSZB722	derivative of pAG25 with *ade6-* cloned into BamHI and SalI	this work
pSZB788	pSZB188 with FY29033 *wtf35* cloned into SacI site	this work
pSZB800	pSZB387 with FY29033 *wtf35* cloned into SacI site	this work
pSZB810	pSZB188 with CBS5557 *wtf23* cloned into SacI site	this work
pSZB812	pSZB387 with CBS5557 *wtf23* cloned into SacI site	this work
pSZB849	derivative of pSZB722 that integrates at *ade6* , yielding *ade6* -	this work
pSZB852	pSZB387 with FY29033 *wtf36* cloned into SacI site	this work
pSZB853	pSZB387 with FY29033 *wtf36* cloned into SacI site	this work
pSZB879	pSZB387 with FY29033 *wtf1* cloned into SacI site	this work
pSZB1087	pSZB188 with *Sk wtf11-GFP* cloned into SacI site	this work
pSZB1176	pSZB188 with FY29033 *wtf35-GFP* cloned into SacI site	this work
