# Supplementary material for: Dramatically diverse Schizosaccharomyces pombe wtf meiotic drivers all display high gamete-killing efficiency
Source: PLoS Genet. 2020 Feb 7;16(2):e1008350. doi: 10.1371/journal.pgen.1008350 (PMC7032740; doi:10.1371/journal.pgen.1008350)
Supplement: S7 Table — (PDF) [file pgen.1008350.s017.pdf]

oligo number	sequence (5'-->3')
AO638	GTATTCTGGGCCTCCATGTCG
AO1112	TAACGCCGCCATCCAGTGTCG
PR78	CCTTGACAGTCTTGACGTGC
PR79	CGCACTTAACTTCGCATCTG
588	ATGAGCGAAAAACAGGTTGTAGGGATC
589	GGTACCTGACCTGAATTGTGAGGCCGAGG
590	CCATAGCAGCCAAAAGGGAGGGTTG
591	CACAATTCAGGTCAGGTACCCAACACCCAACTCTCGACTTCCAC
623	CCGGCGATCTAATCTTAAACAAGGT
624	CTTGTTTAAGATTAGATCGCCGGTT
625	AAT GCTTCAAAAT AATTTTGTAA ATCATGTTAT GCCG
626	GTGTCACCTAAATCGTATGTG CGTTGTTCGTTACTATCGCATACACACTC
627	GAGTGTGTATGCGATAGTAACGAACAACG CACATACGATTTAGGTGACAC
628	CCTTCTCGACCTTTTTCGTGGAGACTG AATACGACTCACTATAGGGAG
629	CTCCCTATAGTGAGTCGTATT CAGTCTCCAC GAAAAAGGTC GAGAAGG
630	CCACTTCCCTATCTGCTTGTTTGTTTGTTCTTGTC
631	CGGGATTGCATAG CTTGCAAGGT C
632	CATCTCTACCTTGATTTGACTTTTCTTCCCCTG
634	AATATAGAGCTCCGGGGACGAGGCAAGCTAAAC
645	AATATAGAGCTC CAGTGAAAG TGACATCTTC GTCGGTAGG
646	AATATAGAGCTC AAA GGT AGG AGG GAA CAA GGG GAT TC
656	GTGTCACCT AAA TCG TATGTG CCAGTG TGT TGT GGC GAT ACT TCG
657	CGA AGT ATC GCC ACA ACA CAC TGG CAC ATA CGA TTT AGG TGA CAC
660	GGCGCTTTTAGTGTC GGAATCGTAC
661	TAACATATTACGCTGCCGAAGCTATACC
881	ATATATGAGCTCTGCGGTTTTAAGCTGTTGGC
882	ATATATGAGCTCTACCCATTTTGTCACTGCTCCT
890	GGCAAAGCCCATTTACACGG
891	ATATATGAGCTCTGCGGATCAGTGAATACTAAGCA
975	AATATAACTAGTCAATGATCGTGTCTTGGCTGTGAACAAACATA
976	AATATAACTAGTGCAAGTGAAAAGTAGAATTCCAAAACCCATTT
977	AATATAACTAGTTTCTCTCCATTTTGTTCTGCTTACACCCATCT
978	AATATAACTAGTGTGAATTTAACAAGGCGGAACAACGAGTTTTC
991	ATTATTGAGCTCGGTTAACTCATTCCATTTCGGACCGTATAAAC
1033	ATAGGAGATATCAACGATACATTGAACTGCAGTGACTCTCTTG
1034	ATAGGAGATATCTATAACTGAACTGAGGAACGAGGTTCAGTTGG
1035	ATTATTGAGCTCCGCTCAACTTTCTTATTTAGACACAGATGGGT
1036	ATTATTGAGCTC CATACTCCTCTTGTTTAGGCGACATTCG
1039	ATTATTGAGCTCATCAACACGATAGCATGAGACCTTCTTACCAT
1129	AATATAGAGCTC AAGCCGCACTACAATAGGCTGTTTATAAGAGT
1130	AATATAGAGCTC GGATATGCCAACTGTAAACAATGATCACATCC
1131	AATATAGAGCTC TGATACATTGTGTTCAAAGCCGCTGTTAGTTC
1132	AATATAGAGCTC TCGCATTCTATCTATTCGCTTTTCAGCGTC
1133	AATATAGAGCTC CACCCTAACTTAGGCTTCCTTTATGCATCATT
1134	AATATAACTAGT TAATGTTGCTCACATAGGCCTTAACGTTAACG
1135	AATATAACTAGT TTAAGGAACAGTAAAGCGTAGCTTTATTCGGG
1154	AGCATTGTAACTTCACTTACCAGATGATAC
1202	ATTATTGAGCTCCGTTCTGAGTCACAATATTTTATTATGCTATTC
1346	ATTATT GAGCTC AGTTGTGAATACAACAAGGAGGAACAGCG
1348	ATTATT GAGCTC TGCTTGTACTGCTGTTCGTAATCGCAG
1349	ATTATT GAGCTCTCATTAGTTGATGATAACAGTCAATTAGTGAACTGC
1351	ATTATTGAGCTC TAACAAGGCGGAACAACGAGTTTTCC
1352	ATTATTGAGCTC ATTGGAAAACTCGTTGTTCCGCCTTG
1359	ATTATTGAGCTC AATAACTGGCATGTCATCAGACAGAGAGAG
1360	TAAACCAGCACCGTCACCGCCATATATACTATTCATATATATTCCA
1361	TGGAATATATATGAATAGTATATATGGC GGTGACGGTGCTGGTTTA
1362	ATTATT GAGCTC CAGCAACACAATTACTTTACGTAGCGAGCAG
1365	ATTATT GAGCTC CACAGGTAATATGGGTAACAGGTAATGCTG
1366	TAAACCAGCACCGTCACCGTTTCTATTTAGGATATCTAGTTTGGTAAGTG
1367	CACTTACCAAACTAGATATCCTAAATAGAAAC GGTGACGGTGCTGGTTTA
1368	TAAACCAGCACCGTCACC AACTAGGTCTTCGGGACTTGTTTCATTCAATTG
1369	CAATTGAATGAAACAAGTCCCGAAGACCTAGTT GGTGACGGTGCTGGTTTA
1557	ACCATGCCAGAATGTCCAGG
1565	TTTAGTTACACTGCTTTACTTCTTTATTTT
1566	GTGTCACCTAAATCGTATGTGTTGGACGAAAAATATAAGATTAGATCACAGAATTGCAAG
1567	CTTGCAATTCTGTGATCTAATCTTATATTTTTCGTCCAACACATACGATTTAGGTGACAC
1568	AAACTTCTCCTACTCACAGTCAAGGAGTTTATTTTTTAAAATACGACTCACTATAGGGAG
1569	CTCCCTATAGTGAGTCGTATTTTAAAAAATAAACTCCTTGACTGTGAGTAGGAGAAGTTT
1570	GATAAGAGGGAAGATCGCGTTAGTCAATTC
1571	TGTTTAGGAATTTCCAATCGATCGTTTCGCTAAC
1572	AACAAAAACTACCGTTAGGAAAATTTATGTATATCTCAATTG
1585	CAGACTTTTAGACTAAGACCTTTTGCTAAGAACG
1586	CGTTCTTAGCAAAAGGTCTTAGTCTAAAAGTCTG
1592	ATTATT GAGCTC CGCAGAATCTTTCAAGCAATGCACTTGC
1593	ATTATT GAGCTC CCAAGAAGGGAATGTTCATGCTATCCATG
1635	TATAAGGACGCTTTCTTTAAGGATTATGGTGCAGCA
1636	GCACTTTTGCCAACTTAGCCAACGTACGCTCG
1637	GAACATTGCCCATAATAAATAACACAGAGG
1649	CAGTAGTTCACCAGTTTTCTGTTAGCGAAA
1650	ACCGGCGCATTTTTTGATCAACCTCTTTTTATCGCTCATTCGCCGGGTCACCCGGCCAGC
1651	GCTGGCCGGGTGACCCGGCGAATGAGCGATAAAAAGAGGTTGATCAAAAAATGCGCCGGT
1655	CTGTCGATTCGATACTAACGTAGTTTCATTATTGCTTGGTACTGTCAATAGTCGCGAAGC
1656	GCTTCGCGACTATTGACAGTACCAAGCAATAATGAAACTACGTTAGTATCGAATCGACAG
1657	ATAGTGCGTTTTAGCATGTGGAGGC
1658	AGGTTTAAACAAACGGAGATGTAGAAGATG
1667	TTAGAAGAAAGAGTGGAAGTTTAACGAAGT
1668	ATAAATCTAATTGAACTTACAAGTTATCCTTTCCTTGAAACGCCGGGTCACCCGGCCAGC
1669	GCTGGCCGGGTGACCCGGCGTTTCAAGGAAAGGATAACTTGTAAGTTCAATTAGATTTAT
1670	CTGTCGATTCGATACTAACGTAGCATTCTCGTTATGAATAAGATCACTAAACAAGTTGCG
1671	CGCAACTTGTTTAGTGATCTTATTCATAACGAGAATGCTACGTTAGTATCGAATCGACAG
1672	AGCATATACAATAAGCGACATGTTTGGTTC
1705	TCCAAGAGGAGTAATGCCTAATGGATTGAT
1706	GCATCGATTTTTATCACAATGCTTCCCACC
1707	AGTATAGTACAATTCCATGTCTCACTTACGCA
1709	TATCCAAAAAGGACCAAAGTTTCACCATCA
1710	GCCAACCCCATAGATGAACAAATAAGAAAAC
1711	GACCCTTTGCGGTGATTTAATGTTTTCAAA
1842	CTGGTCGCTATACTGCTGTCG
1843	GGTCGCTATACTGCTGTCGATTC
2152	GCTCCCATTGAGGATTCTGCAG
2153	GGGCGAACTTCGTCCAACTTTC
2154	CAGCAATTACGTTCCCTTGACAAG
2155	CTCCCATGCAACATACCACCGC
2156	GAGCGTGTCATTGGTGTTATGC
2157	GCTCACCGCGCTTAGAATCG
2158	CTCCCTATAGTGAGTCGTATTGTATCTGTGTTAACAATATTTTGGGCTGTGG
2159	CCACAGCCCAAAATATTGTTAACACAGATACAATACGACTCACTATAGGGAG
2165	GGAAGTCGAGAGTTGGGTGTTGAGGCCTTGACCTGAATTGTGAGGC
2166	GCCTCACAATTCAGGTCAAGGCCTCAACACCCAACTCTCGACTTCC
2210	TAAACCAGCACCGTCACC GAATTCATCTGAATCTTCAAGAAATTACACC
2211	GGTGTAATTTCTTGAAGATTCAGATGAATTCGGTGACGGTGCTGGTTTA
2260	ATATATACTAGTTCATTTGGCTCCTTCTTGTTGT
2261	ATATATACTAGTTGTGCCTCTAATGATCTAGCGT
2287	CATCAAGATCATTCTTTATTTAATAAAACACATGCGCG
2371	ATGAACCCCGAAAGTGAAGCCGGTGACGGTGCTGGTTTAATTAAC
